# Biobehavioral Insights into Adaptive Behavior in Complex and Dynamic Operational Settings: Lessons learned from the Soldier Performance and Effective, Adaptable Response Task

**DOI:** 10.3389/fmed.2017.00217

**Published:** 2018-02-05

**Authors:** Amy J. Haufler, Gregory F. Lewis, Maria I. Davila, Felipe Westhelle, James Gavrilis, Crystal I. Bryce, Jacek Kolacz, Douglas A. Granger, William McDaniel

**Affiliations:** ^1^National Security Analysis Department, Applied Physics Laboratory, Johns Hopkins University, Laurel, MD, United States; ^2^BrainBody Center for Psychophysiology and Bioengineering, Department of Psychiatry, University of North Carolina, Chapel Hill, NC, United States; ^3^Intelligent Systems Engineering, Indiana University Bloomington, Bloomington, IN, United States; ^4^Kinsey Institute, Indiana University Bloomington, Bloomington, IN, United States; ^5^Gavrilis Research Group, Alexandria, VA, United States; ^6^T. Denny Sanford School of Social and Family Dynamics, Arizona State University, Tempe, AZ, United States; ^7^Institute for Interdisciplinary Salivary Bioscience Research (IISBR), University of California, Irvine, CA, United States; ^8^School of Nursing, Johns Hopkins University, Baltimore, MD, United States; ^9^Bloomberg School of Public Health, Johns Hopkins University, Baltimore, MD, United States; ^10^School of Medicine, Johns Hopkins University, Baltimore, MD, United States

**Keywords:** adaptability, resilience, problem solving, military-relevant challenge, leadership, autonomic regulation, heart rate variability, electrodermal activity

## Abstract

The purpose of this study was to explore the biobehavioral correlates of adaptive behavior in the context of a standardized laboratory-based mission-relevant challenge [the Soldier Performance and Effective, Adaptable Response (SPEAR) task]. Participants were 26 healthy male volunteers (M = 34.85 years, SD = 4.12) with active military duty and leadership experience within the last 5 years (i.e., multiple leadership positions, operational deployments in combat, interactions with civilians and partner nation forces on the battlefield, experience making decisions under fire). The SPEAR task simultaneously engages perception, cognition, and action aspects of human performance demands similar to those encountered in the operational setting. Participants must engage with military-relevant text, visual, and auditory stimuli, interpret new information, and retain the commander’s intent in working memory to create a new plan of action for mission success. Time-domain measures of heart period and respiratory sinus arrhythmia (RSA) were quantified, and saliva was sampled [later assayed for cortisol and alpha-amylase (sAA)] before-, during-, and post-SPEAR. Results revealed a predictable pattern of withdraw and recovery of the cardiac vagal tone during repeated presentation of battlefield challenges. Recovery of vagal inhibition following executive function challenge was strongly linked to better task-related performance. Rate of RSA recovery was also associated with better recall of the commander’s intent. Decreasing magnitude in the skin conductance response prior to the task was positively associated with better overall task-related performance. Lower levels of RSA were observed in participants who reported higher rates of combat deployments, and reduced RSA flexibility was associated with higher rates of casualty exposure. Greater RSA flexibility during SPEAR was associated with greater self-reported resilience. There was no consistent pattern of task-related change in cortisol or sAA. We conclude that individual differences in psychophysiological reactivity and regulation in response to an ecologically valid, military-relevant task are associated with performance-related adaptive behavior in this standardized operational setting. The implications for modern day warfare, where advancing our understanding of the nature of individual differences in adaptive problem solving is critical to mission success, fitness for duty, and other occupational health-related outcomes, are discussed.

## Introduction

Modern day warfare requires real-time problem solving to meet changing uncertainties in operational environments. The US military’s experiences over the last two decades have illuminated the changing nature of conflict. Adversaries are enabled with technology, lethality has increased over time, and the battlefield is non-contiguous and decentralized. Warfare is more complicated, fast paced, requires less kinetic action, and more often involves the integration of security operations with development programs relative to past eras of conflict. Soldiers and small unit leaders must be resourceful, creative, and adaptively recalibrate to negotiate and succeed on the modern battlefield (1, 2).

The Army’s Warfighting Challenge #10 (i.e., first-order problems critical to future capabilities) is to “develop agile, adaptive, and innovative leaders who thrive in conditions of uncertainty and chaos, and are capable of visualizing, describing, directing, leading, and assessing operations in complex environments and against adaptive enemies” (3). As such, *adaptability* is a core capability and is critical to mission success, fitness for duty, and is likely related to other occupational health-related outcomes (e.g., hypertension, sleep disturbance, and depression).

Adaptive leadership involves selective attention to key components of events and the significant information streams linked to those events, while minimizing attentional division caused by irrelevant back-or foreground stimuli. Adaptive leaders are calm and thoughtful under pressure. They are also sensitive to their changing surroundings, absorb what is going on, and comprehend the environment, the situation, and the circumstance. Adaptive leaders also understand and interpret broad high-level strategic mission goals and create and execute action plans that are appropriate for the unique circumstances encountered at the tactical level. They can recalibrate their actions (i.e., fighting vs. negotiating vs. humanitarian assistance) efficiently and effectively, and can sustain high degrees of adaptability across mission duration (4).

Given the dynamic, uncertain and real-time performance requirements in which soldiers need to be able to think, know, respond and do, our research team has proposed a working model that characterizes *adaptability* with respect to the biological, cognitive, and affective resources that are managed in coordination to achieve optimal performance under challenge (5, 6). We anticipate that optimal performance under complex, chaotic, dynamic conditions involves the coordinated reactivity and regulation of environmentally sensitive behavioral, cognitive, and physiological processes (7–9). At the measurement level, our model includes individual differences in executive function (e.g., cognitive inhibition, cognitive flexibility, and working memory) (10, 11) as well as in the flexibility, reactivity, and regulation of the two main components of the psychobiology of the stress response—the autonomic/sympathetic nervous system (ANS/SNS) and hypothalamic–pituitary–adrenal (HPA) axis (7, 12–23). A substantial literature supports the interpretation that individual differences in the HPA response to challenge reflect a “defeat” response, whereas activation of the ANS/SNS to challenge reflects a “defense” response (24). That is, increases in cortisol (the primary product of HPA axis activation) are common when individuals are confronted with unfamiliar circumstances and they are overwhelmed, withdraw, and exhibit high degrees of distress. By contrast, increases in ANS/SNS activation are more likely when individuals experience physiological arousal and are mobilizing that arousal to “fight or flight”—attentional focus, cognitive effort, and taking action-oriented steps in an effort to rise to the challenge.

A central challenge to studying adaptability in the modern American warfighter is the necessity to evaluate inter- and intra-individual differences in a social ecologically valid but standardized setting outside of the operational theater. In an effort to address this challenge, Haufler et al. (6) created the Soldier Performance and Effective, Adaptable Response (SPEAR) task. SPEAR emulates the time-demand, multisensory, multitasking, tactical battlefield challenges requiring adaptive decision making. This 90 min task includes two distinct components/modules consisting of 18 independent embedded mission challenges each. The task was designed to simultaneously engage perception, cognition, and action aspects of human performance like those encountered in the operational setting. Participants engage with text, visual, and auditory stimuli, interpret new information, retain the commander’s intent in working memory while calling up training and experience information from memory stores, integrate new experiences with learned behaviors, and compose a response to report their leadership action plan (i.e., adaptive response to the dynamics of the mission challenge). To complete the SPEAR task, the participant must efficiently and effectively negotiate the back and forth of problem engagement, solution development, and dissemination of the action plan, across multiple back-to-back trials. In this manner, the SPEAR task reflects the tactical leader’s operational requirement to be able to sustain a psychophysiological response that is supportive of meeting a new challenge, demonstrating adaptive leadership and recovering in order to prepare for the next challenge.

The purpose of this preliminary study was to begin to explore the biobehavioral correlates of adaptive behavior in the context of this standardized laboratory-based mission-relevant challenge task. Participants were 26 healthy male volunteers (M = 34.85 years, SD = 4.12) with active military duty and leadership experience within the last 5 years that included multiple leadership positions, operational deployments in combat zones, had interactions with civilians and partner nation forces on the battlefield, and had experience making decisions under fire. Time-domain measures of heart period (HP), respiratory sinus arrhythmia (RSA), and skin conductance were quantified, and saliva was sampled before-, during-, and post-SPEAR. Saliva samples were assayed for cortisol (HPA axis) and alpha-amylase (ANS/SNS). In this group of experienced military leaders, we expected that individual differences in performance on the SPEAR task would be associated with ANS/SNS, but not HPA axis, task-related reactivity and regulation, as well as with executive function.

## Material and Methods

### Participants

The participants for this study were qualified volunteers recruited from Fort Meade, MD, USA, The Johns Hopkins University Applied Physics Laboratory, and the greater Washington DC metropolitan area. Volunteers had to have served on active duty in the military within the past five years, held at least one leadership position in any of the following ranks: E-5 (SGT), E-6 (SSG), E-7 (SFC), E-8 (MSG), O-3 (CPT), or O-4 (MAJ), and served in an Army Combat Arms Military Occupation Specialty (MOS). The final study sample consisted of 26 healthy male participants aged 27–41 years (M = 34.88; SD = 3.73) who held multiple leadership positions, had multiple operational deployments in combat, had interactions with civilians and partner nation forces on the battlefield, and had experience making decisions under fire. The participants had high levels of civilian and military education, exercised regularly, and were from combat arms branches to include Infantry, Armor, Artillery, Special Forces, Engineers, and Aviation branches. This study was carried out in accordance with the recommendations of the Johns Hopkins Medical Institution Review Board. This protocol was approved by the Johns Hopkins Medical Institution Review Board.

### Apparatus and Measures

The laboratory was configured with two stations each equipped with a Dell laptop personal computer (PC), display monitors, keyboard, and mouse to support the experiment. The experimenter station was used to initiate the experimental protocol and monitor data acquisition. The participant station was configured for the participant to engage with and complete the experimental protocol. Participant responses were acquired usingthe PC keyboard and the five-button Chronos^®^ response box. Koss QZPro Noise Cancellation Sterephone headphones were worn by the participant during the auditory discrimination baseline task and the Soldier Performance and Effective, Authentic Response (SPEAR) adaptability test task. A BIOPAC Systems, Inc. wireless data acquisition system consisting of the MP150 data acquisition hardware (Ethernet-ready data acquisition analysis unit), UIM100C (universal interface module used to connect amplifier modules and signal cables to the MP150), STP100C (safely isolates digital inputs and outputs to and from the MP150; connects the MP150 to the PC running the assessment applications), dual wireless respiration and electrocardiography (ECG) BioNomadix module pair (BN-RSPEC), and electrodermal activity (EDA) BioNomadix module pair (BN-PPGED). Cardiovascular and EDA data were acquired using BIOPAC Systems, Inc. AcqKnowledge software version 4.4. Biophysical data were collected continuously at a sampling rate of 1,000 Hz. Psychology Software Tools, Inc. E-Prime 2.0 (E-Prime) was used to program theexperimental protocol as presented to the participant to include all baseline, executive function and SPEAR task events. Inline code was written in the E-Prime study protocol to designate an event mark (±5 mV excursion) in the AcqKnowledge physiological recording to indicate the onset and offset of specific stimuli events across the study protocol. Absorbent 1 × 4 cm polyolefin swabs (SalivaBio, Carlsbad, CA, USA) were used to obtain the saliva samples. Immediately after collection, saliva samples were stored in a 3.2-cubic-foot Danby upright freezer and samples remained frozen until the day of assay. All saliva samples were assayed for cortisol (Cortisol, CAT# 1-3002) and alpha-amylase (sAA, CAT# 1-1902) using commercially available immune or kinetic assay protocols without modification to the manufacturers recommended instructions (Salimetrics, Carlsbad, CA, USA).

### Behavioral Assessments

#### Demographic, Military Experience and Mental Health Self-Report

Demographic questions consisted of age, gender, education (general and military), race, ethnicity, and physical fitness. Participants also provided information about their rank, military occupation specialty, military training, military deployments, and leadership experience. Measures of mental health were collected to include the Profile of Mood States (POMS-2), Positive Negative Affect Scale (PANAS), State-Trait Anxiety Inventory, and the Dispositional Resilience Scale (DRS).

The POMS is a measure of relatively recent mood state elevations, referred to as Total Mood Disturbance, and differentiates between six clinically important mood state dimensions including fatigue, tension, depression, anger, confusion, and vigor (25, 26). The POMS-2, employed in the present study, also includes a scale for friendliness.

The Positive and Negative Affect Schedule (PANAS) is used to assess current and average human emotion along positive and negative dimensions. PANAS is most commonly used to show relations between positive and negative affect with personality states and traits (27).

The State-Trait Anxiety Inventory for Adults is used to measure levels of anxiety, with separate measures for a person’s general trait level, as well as the short-term effect of the state or anxiety at that particular moment (28, 29).

The DRS is an assessment of hardiness which is defined as a resilient stress response pattern (30–33). Hardiness is composed of three sub-components, namely commitment, control, and challenge. Commitment reflects an individual’s interest in the world, and the degree to which they believe events have meaning. Control reflects the degree to which an individual believes that they can influence the events occurring around them. Challenge reflects an individual’s disposition toward change and new experiences. The DRS-15 consists of 15 questions and was used in the present study.

#### Baseline Assessments

Three computer-based assessments were completed to establish physiological and performance baseline responses. The first baseline task was a cardiovascular challenge consisting of a sequence of sit and stand periods from which the cardiovascular response to the postural change was used to relate nervous system flexibility to individual physiological adaptability. The initial sit period was 2 min in length, followed by five alternating stand/sit periods each for 1 min in length, and a final 5-min sit period. The second initial assessment was a two-choice reaction time (RT) task (34, 35) which was administered to confirm participants could generate a typical choice RT response and for use as referent data for analysis of the Ericksen-Flanker task. Participants were prompted with either a “1” or “2” stimuli to which they responded as quickly as possible by pressing the corresponding “1” or “2” on the Chronos response box. The stimuli period was 1 s, and the response period was 3 s, with a variable 1–3-s intertrial interval. The choice RT task took a total of 5 min to complete. The third initial assessment was an auditory discrimination task [similar to an auditory odd-ball task (36)] in which participants wore headphones and low and high tones were presented (80/20 ratio, low/high) for 1 s with a variable intertrial interval of 3–5 s. The participant was asked to count the high tones and report the final count number upon completion of the baseline assessment. The series consisted of a total of 60 tones with a delay of 3–5 s between each tone. Seventy-five percent of the tones (45 trials) were low, and 25% of the tones (15 trials) were high. This task took a total of 4 min to complete. This task was a check to assure the participants could distinguish the low and high tones and practice discriminating between the two, in preparation for the dual task requirements of the SPEAR task.

#### Tests of Executive Function

Three tests of executive function were administered *via* computer to measure cognitive inhibition, cognitive flexibility, and working memory (10, 11). The three tests were the Eriksen Flanker, the Iowa Gambling Task (IGT), and the N-back task (37–44). The Eriksen-Flanker is a test of cognitive inhibition that examines the distractor interference effect from congruency (37). Research shows that attentional control processes in the Eriksen-Flanker are influenced by congruency sequence effects (i.e., the Gratton effect), manifesting as a smaller flanker interference effect after incongruent trials. These results inform the contribution of target flanker and response repetition (38, 39). Participants completed 256 trials that were presented in a balanced, pseudo-randomized design. Specifically, each pattern was displayed for 0.8 s. A fixation cross was displayed for 1 s between trials. The response window for each trial included the intertrial fixation period. The trial structure was generated such that there was an even distribution of trial types [25% Congruent, directed to the right (C), 25% Congruent, directed to the left (C), 25% Incongruent, directed right (I), 25% Incongruent, directed left (I)], congruencies (50% C, 50% I), and previous trial to current trial congruencies (25% II, 25% IC, 25% CC, 25% CI) (39). Participants were given 12 practice trials prior to starting the task. The duration of the task was approximaely 10 min.

The IGT (40) consisted of 200 trials. The goal of the task is to maximize profit while minimizing loss on a loan of virtual money. The participants were shown four decks of cards face down and given a virtual endowment of $2,000. Every card represented a dollar amount, positive, negative, or 0, that would change their total winnings when selected. For each trial, the participants drew a card from one of the four decks, attempting to maximize winnings and minimize loss. The participant is free to switch from any deck to any other deck at any time and as often as they wish across the task period. The decks provide different levels of fixed rewards and punishments. Two decks provide net winnings, while the other two are net losers. After making a selection, participants were shown a feedback display [6 s (41)] of the reward (wins) and punishment (losses) of their selection on that trial as well as their remaining total amount of money. The participants were given an infinite amount of time to make a selection. Participants were not given practice trials for this task. The duration of the entire task was about 12 min.

The N-back task (tested as the 2-back) (42–44) consisted of 75 trials. Participants were required to monitor a series of stimuli and to respond whenever a stimulus was presented that was the same as the one presented n-trials previously, where *n* = 2 in the study’s version of the experiment. All participants received trials in the same, pseudo-randomized order. The participants were required to monitor a series of letters and to respond with a 1 if the letter was the same as the one presented two trials previously and a 2 if the letter was not the same as the one presented two trials previously. Each letter displayed for 0.5 s. A blank screen displayed for 2 s between trials. A response could be made from the time the stimuli appear to the end of the 2-s blank screen period. Participants were given 12 practice trials prior to starting the task. The duration of the task was approximately 6 min.

#### Soldier Performance and Effective, Authentic Response (SPEAR) Task

The SPEAR task was developed to test adaptive decision-making in response to authentic military scenarios based on the Army’s definition of adaptability and the study’s operational definition of soldier adaptability (6, 45, 46). The SPEAR task was a computer-based task consisting of two blocks of 18 trials each, for a total of 36 trials. Other computer-based tasks, like SPEAR, have been shown to evoke useful measurable responses in decision based training and testing applications (47–50) without compromising authenticity. The block and trial structure was fixed. Each block began with instructions followed by strategic context, mission statement, and commander’s intent. Eighteen trials were then presented to closely approximate tactical challenges relevant to the block’s strategic context. Participants were expected to retain (increase working load and working memory) the overarching objectives and goals throughout the test and were expected to develop creative ways and new approaches to achieve the commander’s intent in the face of changing situations and obstacles to the mission when engaging with the 18 trials in each block. In one block, the mission and commander’s intent focused on combat operations (CO) and in the other the mission and commander’s intent focused on security force assistance (SFA). These two characterized much of the participant pool’s military experience over the last 15 years in Iraq, Afghanistan, and other combatant command areas. COs scenarios included react to contact, react to IEDs, react to casualties, offensive and defensive actions, patrolling, observation post, combat advising, and security and stability operations. The Security Assistance Force block operations included training, advise and assist, and Coalition operations, Embassy relations, human rights issues, and cultural, regional, and political considerations. The block order was counter-balanced across subjects so that one-half completed the COs block first and the other half competed the SFA block first.

Participants were also required to accomplish a secondary auditory discrimination task, similar to the auditory odd ball paradigm (36), while completing each SPEAR block of trials. The purpose of the auditory challenge was to emulate the multisensory and -dimension stimuli environment similar to that encountered on the battlefield. High and low tones were presented for 1 s, with a variable intertrial interval ranging from 3 to 5 s. Tones for the CO block of trials were presented in an 80/20 (low/high) ratio with a variable intertrial interval of 3–5 s. Tones for the SFA block of trials were presented in an 85/15 (low/high) ratio with a variable intertrial interval of 3–5 s. Participants were instructed to keep a running count of all high tones across each block and had to report their total high tone count at the end of the block. The correct number of high tone counts was 111 for CO and 84 for SFA. At the end of each block, participants were also asked to recall the commander’s intent from the initial instruction set as a working memory check and to begin to understand the relationship between adaptability and knowledge of mission commands.

Each specific SPEAR trial consisted of a fixation cross (3 s), scenario description (30 s), ensemble video clips (30 s), response prompt (10 s), and response period (105 s). Specific videos, images, and text were selected from the Defense Video and Imagery Distribution System (https://www.dvidshub.net), edited, and reviewed to create the desired intent for each trial scenario. The response prompt instructed the participant to respond to the challenge as if they were reporting their action plan to their higher commanders (Up), adjacent units (=), or subordinates (Down), balanced in random order across each block, to provide a level of uncertainty within each trial and to mitigate any tendency of the participant to anticipate the response prompt or respond passively to the stimulus. Participants typed their action plan.

**Figure 1:**
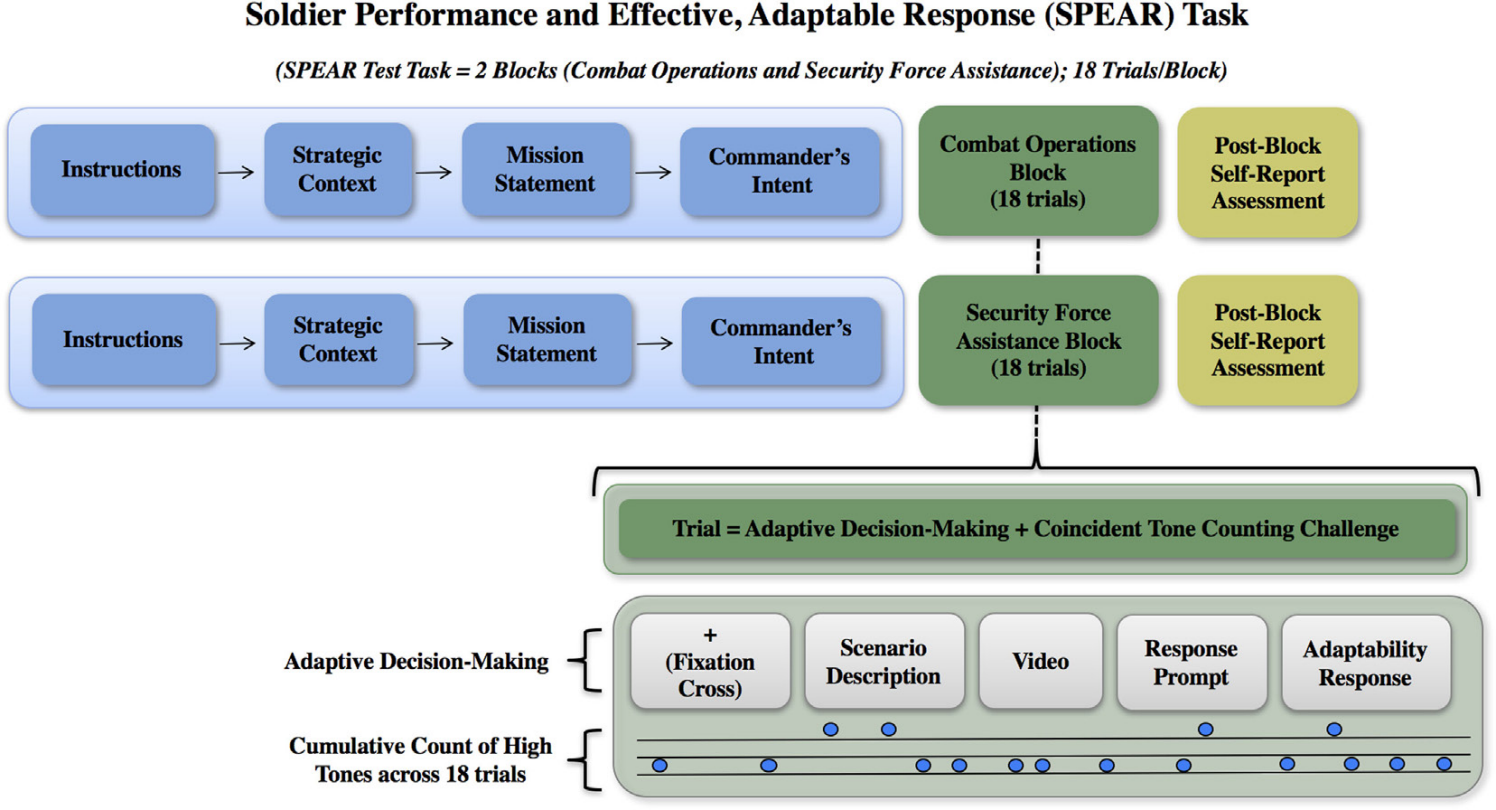
Soldier Performance and Effective, Adaptable Response (SPEAR) task design and trial structure. The initial strategic guidance (i.e., strategic context, mission statement, and commander’s intent) is provided at the beginning of a block of trials is show in the blue boxes. This is provided one time at the beginning of each block of trials. The following 18 trials occur within the context of that initial strategic guidance. The Combat Operations and Security Force Assistance blocks are illustrated as green boxes. The gray boxes illustrate the specific trial elements and auditory discrimination task.

Each of the trials had one of two stimuli type, consistent (C) or inconsistent (IC), that were presented nine times each in a pseudo-randomized order over the course of each block. A consistent stimulus presented a difficulty to task completion that could be considered “routine” or that might be expected to occur in the accomplishment of the mission task. An inconsistent stimulus presented a challenge to completing the task, one that may have required modifying the task or abandoning it all together in order to achieve the purpose of the mission statement and meet the commander’s intent. Inconsistent trials included a higher magnitude of change in the situation and environment, and required a higher level of adaptability to achieve the commander’s intent. This aspect of the design provided varying levels of challenge to measure adaptive performance within the test. See Figure 1 for an illustration of the SPEAR task design and structure.

A scoring rubric (51, 52) was developed to evaluate the participants’ responses provided during the SPEAR task. The rubric consisted of an eight category scale in which each category represented a dimension of adaptability ranging from basic recognition of an altered situation to demonstration of an action plan that meets the commander’s intent and meets the trial-tactical situation. A participant could earn one point for each category met with a range of possible scores for each trial of 0–8. The participants’ responses were scored independently by three trained raters who were military, operations and system engineering subject matter experts. Interrater reliability was checked by computing the intraclass correlation (ICC) coefficient (M = 93.1%, SD = 1.87%) and the Cronbach’s Alpha (M = 94.2%, SD = 1.39%).

### Procedures

**Figure 2:**
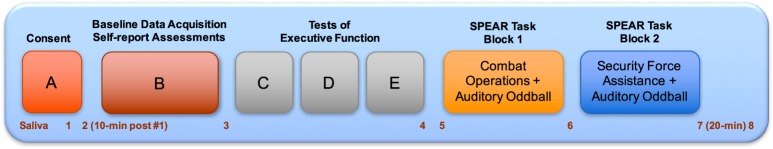
Adaptability protocol. Task events are illustrated and saliva sampling frequency noted. Obtaining the informed consent **(A)** was followed by completion of self-report assessments, instrumentation and the baseline assessments **(B)**. The Ericksen-Flanker, Iowa Gambling Task, and 2-Back tests of executive function were then administered [**(C–E)**, respectively]. The Soldier Performance and Effective, Adaptable Response (SPEAR) task Combat Operations and Security Force Assistance blocks of trials were then completed (orange and blue boxes). Following each of the tests of executive function and both SPEAR blocks of trials, the participant sat quietly for 2-min for a post-task recovery period. The saliva sample acquisition schedule is noted at the bottom of the figure in red text.

Testing was administered in a laboratory environment at The Johns Hopkins University Applied Physics Laboratory (JHU/APL). See Figure 2 for an illustration of the protocol. On arrival, the participant was asked a series of questions to confirm eligibility, then read and provided informed consent. The first saliva sample was taken after participants provided informed consent and then periodically (for a total of eight samples) across the testing session. Saliva sample 2 occurred 10-min post-sample number one. After participants completed the demographic and other self-report assessments, they were instrumented with the ECG and EDA sensors. Participants completed the sit-stand, RT and auditory baseline tasks during which all data were collected and signal quality was confirmed. Saliva sample 3 was obtained immediately following the set of baseline tasks.

The tests of executive function were administered in the following order: Ericksen-Flanker, IGT, and 2-Back. Saliva sample 4 was obtained immediately following completion of the 2-Back. Participants were given a 10-min break prior to starting the SPEAR task. Participants completed three practice trials of the SPEAR task and received feedback after each of their responses to assure they understood the instructions, timing, and requirements of the task. Saliva sample 5 was acquired at the completion of the practice trials and prior to the start of SPEAR test task. Following each block of SPEAR trials, participants completed a post-block survey to include ratings of arousal, mental effort and emotion valance [*via* visual analog rating scale (53)], and to record their tone count and recall of the commander’s intent. Saliva samples 6 and 7 were acquired just after completing each block of SPEAR trials. A final set of self-report assessments were completed after all SPEAR trials were completed to obtain the participant’s observations on the SPEAR task, identify their referent source for their adaptability responses, and strategy for handling the tone count and adaptability challenge. Participants sat quietly for a 20-min period prior to providing the last saliva sample (sample 8). At the end of the experiment, participants were deinstrumented, debriefed, and escorted to the building entrance.

### Signal Processing

#### Electrocardiogram Signal Processing

Electrocardiogram signals were acquired using a BIOPAC MP150 system with the wireless ECG amplifier module. ECG signals were digitized at 1,000 Hz and stored in a continuous record along with several other channels of information (EDA, and a digital event marker signal). Data were processed within custom-designed Labview software (The Brain Body Center for Psychophysiology and Bioengineering, University of North Carolina at Chapel Hill). The processed data included ECG R-peak detection, interbeat interval (IBI) editing, event marker classification, IBI transformation, and parameter extraction.

The R-wave locations were identified to generate the IBI series for analysis while minimizing artifacts and missing data. ECG quality was evaluated, and the R-wave peak inflection point times were extracted to generate the IBI series sequential R-R intervals data for analysis. Where necessary, preprocessing of the ECG waveform included bandpass filtering and/or inversion of the signal polarity to enhance signal quality. An algorithm was used to fit a second-order polynomial to sequential groups of ECG samples (three to six points). The polynomial fit was tested at each peak against a threshold. Peaks lower than the threshold were ignored. For ECG data with a stable baseline, a single threshold was used through the entire data set. Conversely, for ECG data with variable ECG amplitude and/or quality, an adaptive windowing approach was used in which small temporal windows of ECG were analyzed. Extracted parameters iteratively updated the length and position of the next analysis window (54).

While editing the IBI, any missing R-wave detections, preventricular contractions, other types of arrhythmias, and erroneous peak identifications were removed by trained editors using visual inspection. Motion artifacts, device communication failures, and natural transient physiological events led to a small number of these types of edits for the recordings. An algorithm was used to inspect the trigger channel from the BIOPAC to identify task segments. The event marks generated by E-Prime were used to identify each task by name, start time, end time, and duration. Events and BIOPAC signals were further separated into two segments of the protocol, each containing multiple tasks: the baseline segment and the adaptability task testing segment. Baseline segments consisted of posture challenge, choice reaction-time, auditory discrimination, Eriksen Flanker, IGT, and 2-back recall tasks. Rest periods were observed before and after each of the aforementioned tasks.

To isolate the RSA component of heart rate variability, the team conducted IBI transformation. This was based on the Porges-Bohrer method (20, 54), which employs a time-frequency method to extract a band-limited component from the HP time series that represents RSA across the full duration of each segment (baseline or adaptability task testing). During this step, a 5-Hz time sampled raw IBI signal and a 5-Hz RSA component were created. From these two series, 15-s windows (epochs) of RSA (magnitude of the oscillation) and HP (mean IBI value) were computed. The set of HP and RSA values within each event (e.g., IGT) were then used to calculate the following parameters of nervous system regulation of cardiac function: mean HP and RSA, HP, and RSA change over time, short-term correlation between HP and RSA, and linear regression slope between HP and RSA. An additional set of parameters investigating the relationship between HP and RSA across all recovery period epochs was also quantified.

#### EDA Data Processing

To prepare the raw EDA data for analysis, the data were downsampled to 250 Hz. Ledalab (14, 15, 55) was used to compute the number of skin conductance responses (SCRs), as well as the total sum, average, largest value, and the SDs of the amplitudes, areas, and rise times for each the referenced minute during task engagement. Ledalab provides two types of skin conductance analysis: continuous decomposition analysis (CDA), which produces estimates of skin conductance levels, and discrete decomposition analysis (DDA), which produces estimates of SCRs. The DDA methodology, which produces estimates of SCR’s, was employed in the current study. DDA was used to predict when SCR stimuli occurred through a method of decomposing the skin conductance data into a distinct phasic component and a distinct tonic component by means of *nonnegative deconvolution* (14, 15). Nonnegative deconvolution captures and explores all unique deviations of the general response shape and computes a detailed full model of all components in the entire data set. When the tonic component and the individual calculated SCRs (each with a calculated rise time, amplitude, and area) are combined (summed), the result can be compared to the original data, and an error (difference) can be calculated. When the error level is within a small acceptable threshold, then the predicted stimuli times can be known. This method is especially suited for psychophysiological models in which the SCR response to stimuli engagement is of interest.

#### Collection of Saliva and Determination of Salivary Analytes

Across the task series (see Figure 2 for sample collection timing), eight saliva samples were collected. On each sampling occasion, a 1 × 4 cm oral swab was placed under the participants tongue for 2 min. After collection samples were stored at 4 C until they were frozen (within 15–20 min) at −20 C. All samples remained frozen until the day of assay on which they were thawed to room temperature, vortexed, and then centrifuged for 15 min at 1,500 *g*. Sample volumes were estimated by weight and used to determine saliva flow rate (mL/min). Samples were tested for salivary cortisol using a high sensitivity enzyme immunoassay. The test used 25 µL of saliva per determination, had a lower limit of sensitivity of 0.007 µg/dL, a standard curve range from 0.012 to 3.0 µg/dL, and average intra-assay coefficient of variation (CV) of 5.42%, and an average interassay CV less than 10%. Following Granger et al. (17, 18), all samples were also assayed for sAA by kinetic reaction assay. The test volume was 10 μL of a 1 × 200 dilution, lower limit of sensitivity was 0.4 U/mL, and inter- and intra-assay CVs were on average less than 15 and 10%, respectively. There was no association between salivary cortisol or sAA scores and salivary flow rate. Prior to analyses, cortisol and sAA values that were greater than three SDs from the mean were winsorized. Next, the variables were transformed to meet normality assumptions (natural log and square root transformations for cortisol and sAA, respectively). Table 1 shows the Pearson correlations and descriptive statistics for all salivary biomarkers.

**Table 1:** Correlations among all cortisol and sAA samples.

		1	2	3	4	5	6	7	8	9	10	11	12	13	14	15	16
1	Cortisol 1																
2	Cortisol 2	0.85**															
3	Cortisol 3	0.57**	0.81**														
4	Cortisol 4	0.45*	0.54**	0.68**													
5	Cortisol 5	0.64**	0.65**	0.56**	0.72**												
6	Cortisol 6	0.15	0.34	0.38	0.42*	0.44*											
7	Cortisol 7	0.24	0.47*	0.46*	0.48*	0.47*	0.57**										
8	Cortisol 8	0.34	0.50**	0.60**	0.51**	0.52**	0.39	0.74**									
9	sAA 1	−0.04	−0.05	−0.28	−0.27	−0.09	0.07	−0.16	−0.09								
10	sAA 2	0.07	0.02	−0.15	−0.16	0.02	0.06	−0.13	−0.07	0.83**							
11	sAA 3	0.05	0.01	−0.10	−0.15	−0.01	−0.07	−0.14	−0.11	0.86**	0.75**						
12	sAA 4	−0.04	−0.19	−0.34	−0.17	−0.04	0.02	−0.25	−0.24	0.83**	0.77**	0.86**					
13	sAA 5	0.04	−0.02	−0.16	−0.12	0.11	0.05	−0.17	−0.05	0.86**	0.81**	0.88**	0.87**				
14	sAA 6	−0.18	−0.10	−0.18	−0.18	−0.17	0.16	−0.07	−0.14	0.71**	0.68**	0.69**	0.73**	0.67**			
15	sAA 7	−0.14	−0.21	−0.35	−0.23	−0.21	−0.06	−0.21	−0.12	0.86**	0.71**	0.80**	0.84**	0.75**	0.72**		
16	sAA 8	−0.09	−0.17	−0.33	−0.15	−0.07	0.02	−0.26	−0.21	0.77**	0.64**	0.69**	0.86**	0.70**	0.63**	0.89**	

	Mean	0.22	0.22	0.19	0.16	0.13	0.11	0.12	0.11	101.25	88.38	95.17	94.83	164.95	110.06	154.23	145.57
	SD	0.10	0.14	0.09	0.05	0.04	0.04	0.09	0.05	96.29	70.03	76.73	69.55	154.70	90.11	146.84	130.74
	Min	0.08	0.06	0.07	0.09	0.08	0.05	0.04	0.03	12.80	15.10	10.50	8.50	7.90	0.40	11.50	7.20
	Max	0.40	0.59	0.40	0.30	0.19	0.21	0.40	0.23	347.40	281.80	287.30	232.55	555.00	330.30	583.80	528.70

### Analytic Strategy

Descriptive statistics, correlation analyses (parametric and non-parametric), regression analyses, and analysis of variance were computed using Statistical Package for the Social Sciences (SPSS) statistical software (IBM) v24, 64-bit edition. Regression plots were computed using Microsoft Excel v14.0.7106.5003 (32-bit) for visualization. A significance level of α = 0.05 was used for all statistical tests. Corrected significance levels are reported to address increased risk of Type 1 error due to multiple comparisons.

To examine if changes in hormonal biomarkers were associated with self-report measures, we computed change scores for sAA and cortisol, separately, between all adjacent scores from time 3 to time 7 (i.e., change between 3 and 4; change between 4 and 5; change between 5 and 6; change between 6 and 7). The change scores could be either positive, indicating an increase in the biomarker, or negative, indicating a decrease in the biomarker.

## Results

### SPEAR Task Performance

The mean total SPEAR score was 179 (SD = 25.10). The mean score for the CO block was 93.8 (SD = 15.05) and for the SFA block was 85.19 (SD = 12.88). For the purpose of examining the relationship between the biophysical response and SPEAR performance, the response data and SPEAR scores were also analyzed by the time-ordered blocks completed. The mean and SD for the first and second blocks completed were 92.3 (SD = 14.66) and 86.69 (SD = 14.13), respectively. Paired *t*-tests were computed to test for differences between the time-ordered first and second blocks of the SPEAR task across subjects. Participants performed better on time-ordered block one as compared to block two, *t*(25) = 2.03, *p* = 0.05.

### Executive Function and SPEAR Performance

The dependent measure used to detect differential Ericksen-Flanker and SPEAR performance was response time. The consistency of participant response times for the I-C trial pairings was inversely associated with the total SPEAR score. That is, there was a negative correlation, *r*(25) = −0.39, *p* = 0.049, between the SD of the response time for inconsistent (I) to consistent (C) correct response trial pairings of the Ericksen-Flanker and SPEAR total score. No other associations with executive function tasks (i.e., IGT and N-Back) and SPEAR were observed.

### Cardiovascular Reactivity, Regulation and the SPEAR Task

**Figure 3:**
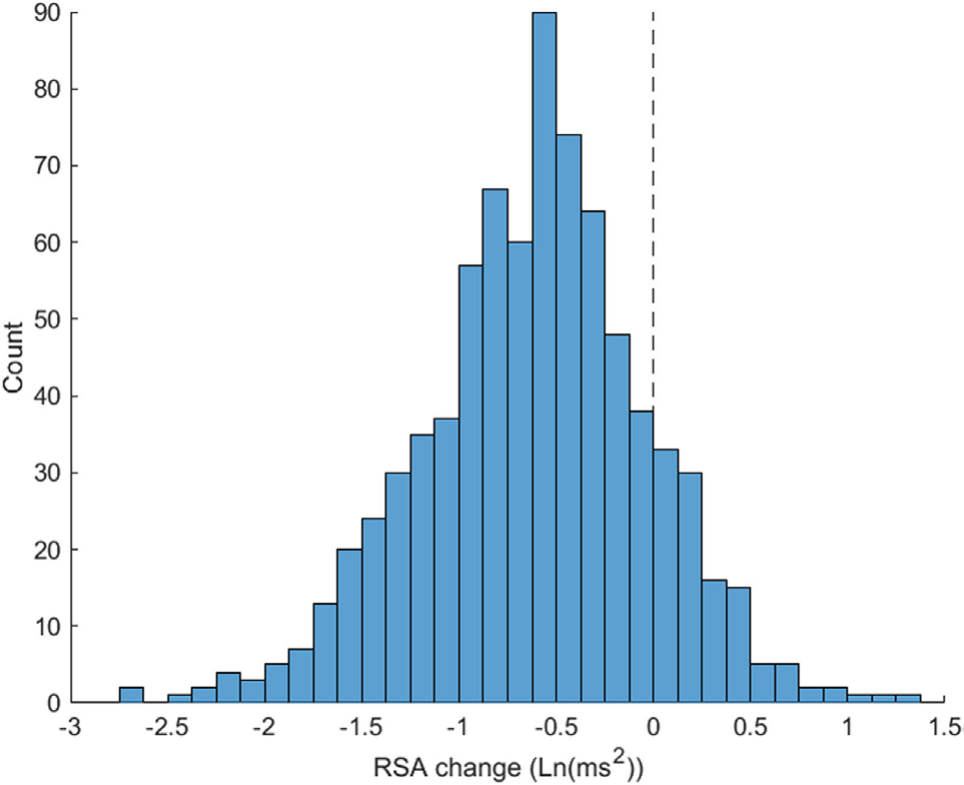
Respiratory sinus arrhythmia (RSA) suppression from Soldier Performance and Effective, Adaptable Response (SPEAR) A to B task phases. Distribution of SPEAR task phases A (Scenario description + Video) to B (Response prompt + Adaptability Response) RSA change scores are illustrated. 86% of trials show RSA suppression from A to B phases (number of trials below 0) indicating shifts in autonomic state to meet “Information Receiving” to “Solution Development” requirements of the task.

**Figure 4:**
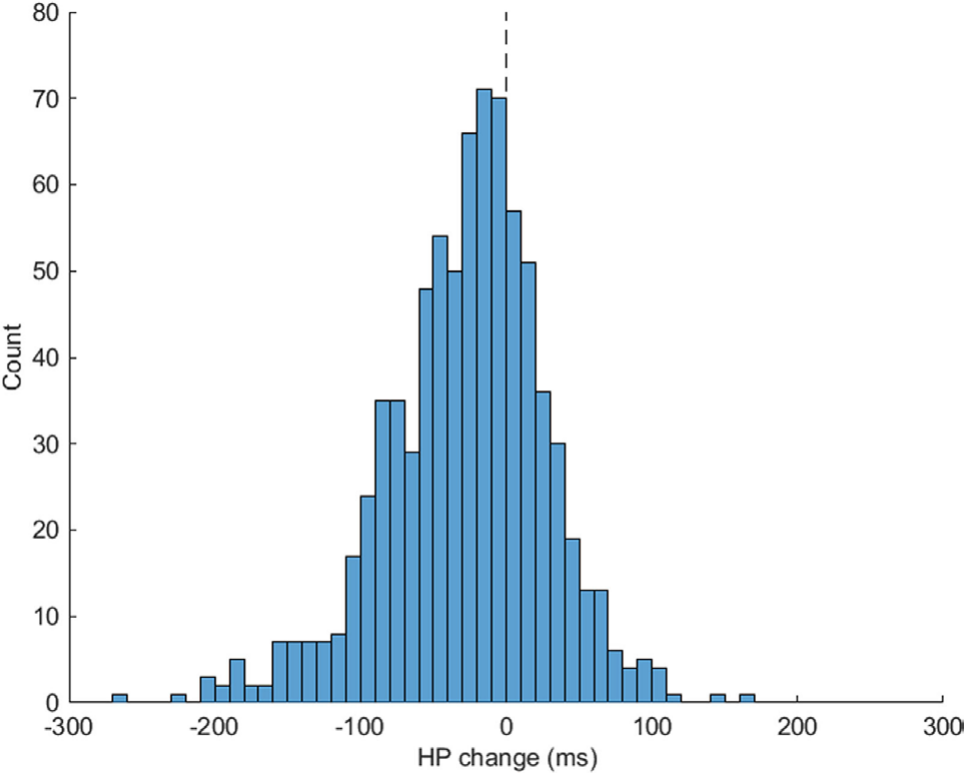
Heart period (HP) change for Soldier Performance and Effective, Adaptable Response (SPEAR) A to B task phases. SPEAR task phases A (Scenario description + Video) and B (Response prompt + Adaptability Response) respiratory sinus arrhythmia change scores are illustrated. 69.6% of the trials show a change in HP from A to B phases (number of trials below 0) indicating shifts in cardiac vagal tone to meet task demands.

Rapid shifts in autonomic state, specifically in cardiac vagal tone as measured by RSA, were observed across the two phases of the SPEAR task. As participants transitioned from the “Information receiving” phase (Scenario description + Video, labeled as “A” phase here) to the “Solution Generation” (Response prompt + Adaptability Response, or “B” phase) phase of each trial, there was a significant decrease in HP of 26.83 ms, *t*(21) = 4.45, *p* < 0.001 and RSA of 0.62 Ln(ms^2^), *t*(21) = 10.51, *p* < 0.001, when comparing average levels across the 36 trials for each subject in each phase. The pattern of RSA suppression was particularly regular (see Figure 3), which shows the distribution of simple change scores across all 36 trials for all 22 subjects. The median change in RSA was −0.58Ln(ms^2^), and 86% of the trials showed a decrease from the A to B phases. A smaller majority, 69.6% of trials, showed a decrease in HP (median change = −21.1 ms, see Figure 4).

**Figure 5:**
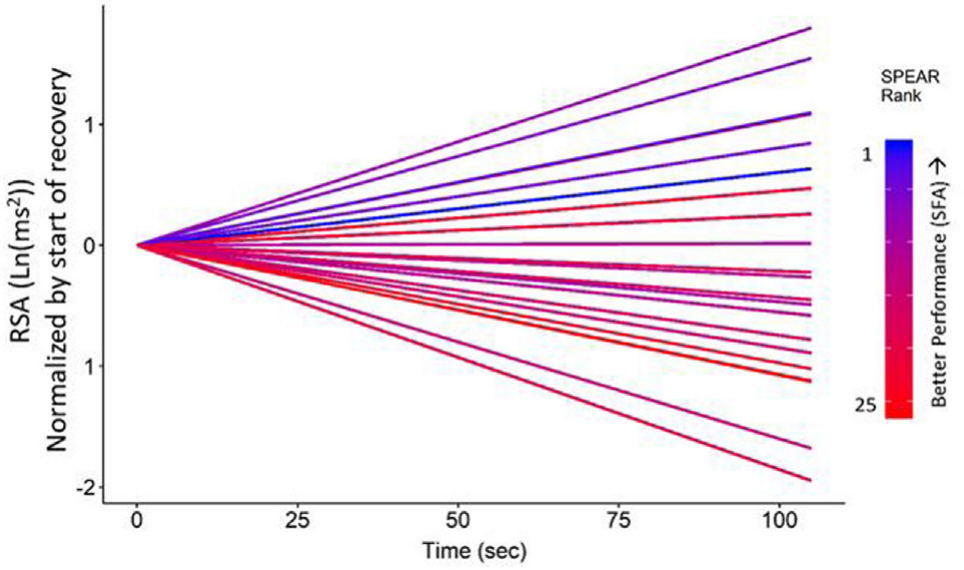
Respiratory sinus arrhythmia (RSA) time trends by Soldier Performance and Effective, Adaptable Response (SPEAR) security force assistance (SFA) ranked score. Recovery of RSA during final recovery baseline prior to the SPEAR task was correlated with greater scores on the SFA block of trials, *ρ*(20) = 0.58, *p* < 0.01. SPEAR performance ranked scores are color-coded on a gradient scale ranging from cool, blue colors (highest scores) to reds (low scores). Highest scores were observed in participants who showed increasing RSA across the 2-min recovery period just prior to the SPEAR task.

While the magnitude of HP and RSA changes were not directly related to performance, the rate of recovery of parasympathetic capacity prior to SPEAR testing was. In the final recovery baseline (FRB) period, prior to the SPEAR tasking (and following the completion of the block of executive function tasks), the rate of RSA change was positively correlated with SPEAR performance and recall of the commander’s intent. Soldiers that showed increasing RSA (i.e., recovered vagal inhibition) demonstrated better performance across the subsequent task, *ρ*(20) = 0.55, *n* = 22, *p* = 0.007, while also having a greater recall of the commander’s intent, *ρ*(20) = 0.47 and 0.62 for the CO and SFA blocks, respectively, *p* < 0.05. This pattern of RSA recovery is visualized in Figure 5. Each line is the slope, shows a subject’s average rate of change in RSA during the FRB (just before SPEAR). The lines are all centered at 0 at the start of FRB for visualization, and the performance on Block 2 is used to color code the lines. The highest scores during SFA tasks are observed in those subjects who showed increasing RSA across the 2-min recovery period FRB, *ρ*(20) = 0.62, *p* = 0.002.

**Figure 6:**
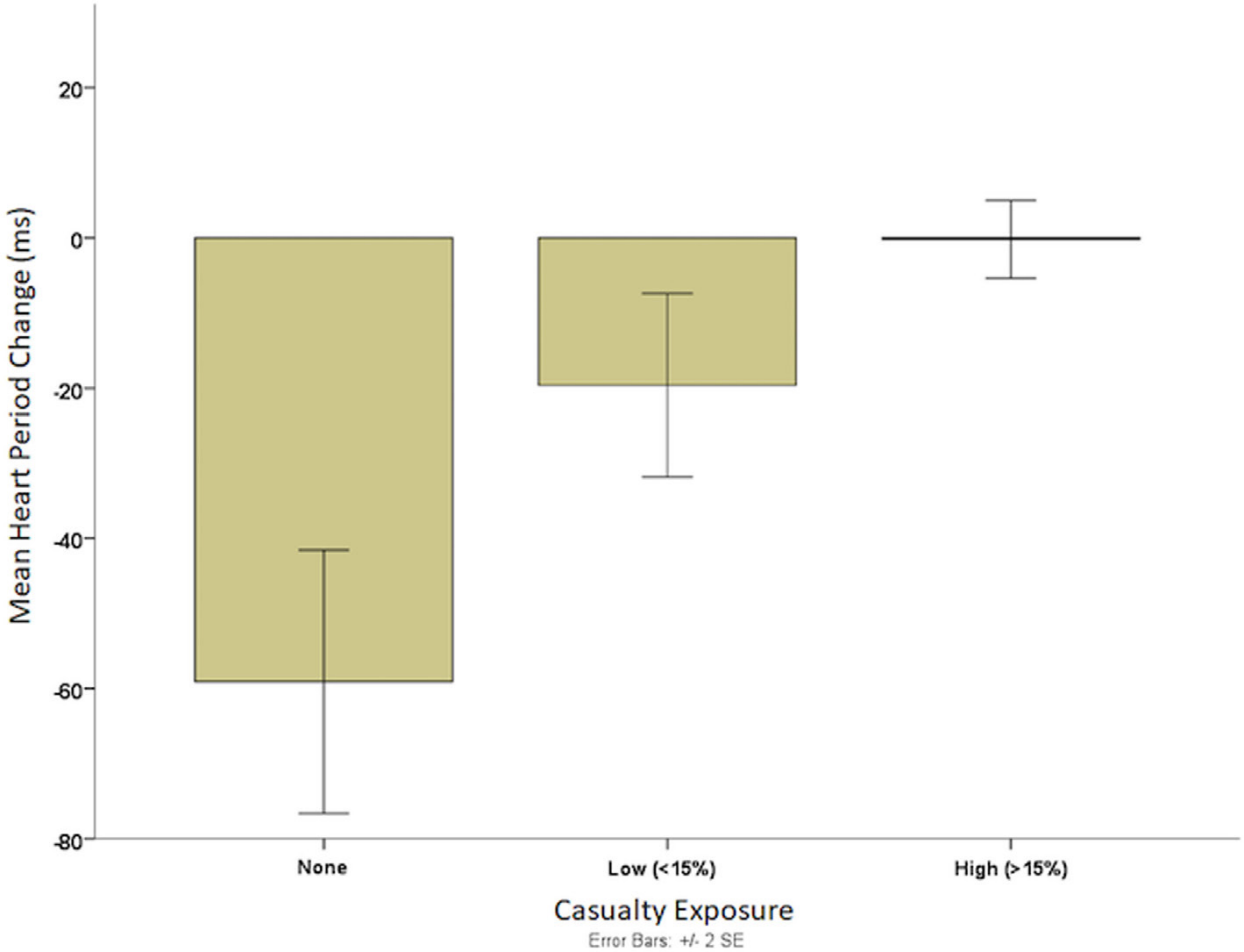
Mean heart period (HP) change during Soldier Performance and Effective, Adaptable Response (SPEAR) task and rates of casualty exposure. A three-group classification derived from the self-reported rates of exposure to combat casualties (1 = none; 2 = 1–15%; 3 = > 15%) is illustrated. The HP value is the change in HP during the SPEAR task. These results illustrate that higher rates of casualty exposure leads to significantly diminished flexibility in cardiac response (less of a saw tooth HP pattern) during SPEAR task engagement.

In addition to performance relationships, HRV parameters were significantly related to self-reported combat experiences. Frequency of COs during deployments was associated with reduced resting RSA levels. The direction of this effect was consistent across several baseline periods with the highest level of significance observed after the sit/stand challenge [*ρ*(22) = −0.47, *p* < 0.05]. Higher rates of self-reported casualty exposure were also related to reduced HP changes when performing the sit/stand challenge, *ρ*(22) = −0.48, *p* < 0.05. RSA reaction to the posture challenge showed a trend toward the same relationship, *ρ*(22) = −0.36, *p* = 0.11. Higher rates of casualty exposure were also negatively correlated with mean HP in several periods. Specifically, shorter mean HP was observed in those with higher rates of casualty exposure after the sit/stand, *ρ*(22) = −0.51, *p* < 0.05 and executive function challenges, *ρ*(22) = −0.50, *p* < 0.05. Casualty exposure was binned into a three-group classification derived from the self-reported rates (1 = none; 2 = 1–15%; 3 = > 15%), and Figure 6 illustrates how higher rates of casualty exposure significantly reduced HP response (less of a sawtooth HP pattern, B − A differences averaged across all 36 trials) during SPEAR task engagement, *F*(2,19) = 7.49, *p* = < 0.01. Greater resilience (on the Commitment subscale) as assessed by the DRS was also related to greater decreases in HP from A to B phases of the SPEAR trials, *ρ*(22) = −0.44, *p* < 0.05.

### Electrodermal Activity

At the recovery period following the block of tests of executive function and prior to beginning the SPEAR task (called FRB), a paired *t*-test revealed a significant difference in the SCR counts in minute 1 and minute 2, *t*(25) = 3.62, *p* < 0.001, indicating a decrease in the number of SCR across that recovery period. Further, less SCR mean area in minute 2 relative to minute 1 (computed as a difference score) at FRB was negatively correlated, *r*(25) = −0.46, *p* < 0.001, with total SPEAR score in the second time-ordered block of trials.

### Salivary Analytes and Adaptability

Since the change score variables violated the normality assumptions and transformations did not improve the skewness of these variables, we examined Spearman correlations between SPEAR total score and both cortisol and sAA change scores, separately. The results showed no significant relation between any of the cortisol change scores and SPEAR total score. One significant relationship emerged for sAA change scores. The change in sAA between time 4 and time 5 was negatively associated with SPEAR total score, *ρ*(25) = −0.41, *p* = 0.04.

## Discussion

In this study of a small group of experienced military leaders, we observed that: cardiac vagal tone demonstrated a predictable pattern of withdraw and recovery during repeated presentation of battlefield challenges, recovery of cardiac vagal tone following a set of executive function challenges led to responses that were more adaptive to the battlefield challenges, and executive function was not directly related to adaptive problem solving capacity. These findings suggest that autonomic regulation plays a critical role in facilitating adaptability and tracking RSA would enhance objective measures of adaptability.

**Figure 7:**
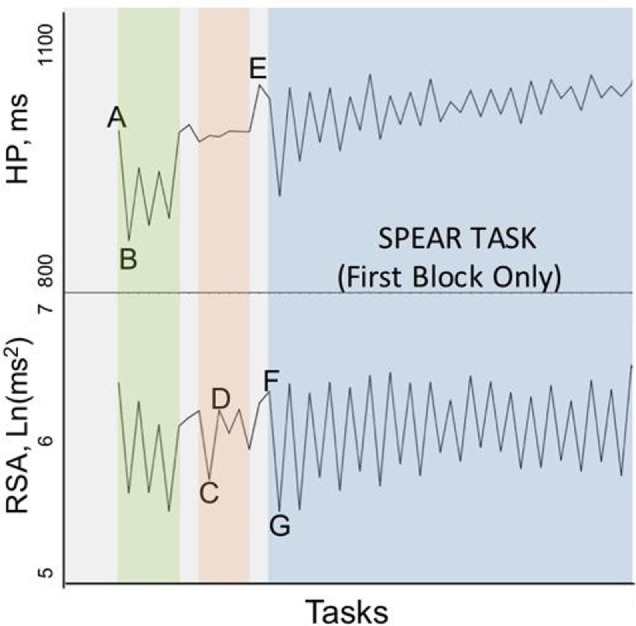
Mean heart period (HP) and respiratory sinus arrhythmia (RSA) cardiac reactivity pattern across the protocol. The mean HP (top) and mean RSA (bottom) over each period in the protocol (A–G) is illustrated. This figure highlights the pronounced cardiac reactivity pattern during the two phases of each trial of the Soldier Performance and Effective, Adaptable Response (SPEAR) task. The first 18 trials of the time ordered SPEAR task are shown to the right of F. The characteristic saw tooth pattern is the heart period (HP) and RSA signal change in response to meeting the demands of the “problem” and “response” phases of the SPEAR task. The regularity persisted in the second time ordered block for RSA but not HP. The tasks notations in the illustration are as follows: A = the initial seated baseline; B = Stand #1; C = Eriksen-Flanker; D = Post Eriksen-Flanker recovery; E = Final recovery baseline (FRB); F = SPEAR Trial 1 (A)—Information Receiving; G = SPEAR Trial 1 (B)—Solution Development.

The clear pattern of withdrawal and recovery observed in the autonomic activity during the SPEAR task highlights the self-regulatory demands of adaptive problem solving (Figure 7). RSA and HP suppression during the transition from “Information Receiving” to “Solution Development” phases of each trial indicate that cognitive processes are demanding different autonomic resources. The vagal break is the quickest means for the body to change arousal state to meet new and demanding situations (22). Rapid recovery of cardiac vagal tone shifts the autonomic system back into normal balance, as at rest, so the soldier is ready for the next problem set. The SPEAR task requires a great deal of cognitive shifting and agile removal of the vagal break and regaining the vagal inhibition. Greater vagal capacity better enables the engagement within and switching between the different demands of the SPEARS task, just as would be encountered on the battlefield.

Furthermore, battlefield experience moderated this pattern of autonomic regulation during the SPEAR task. Specifically, prior exposure to high casualty rates during COs significantly reduced the magnitude of this HP pattern (but not RSA flexibility) during SPEAR task engagement suggesting a reduced self-regulatory capacity or a compensatory reactivity that blunted the impact of cardiac vagal tone on cardiac rate. As such, casualty exposure seems to alter the autonomic nervous system profile by decreasing the efficiency of RSA mediated suppression of cardiac output, which leads to decreased focused attention during SPEAR task engagement and lower levels of adaptive behavior.

The dispositional resilience commitment subscale showed the opposite effect of casualty exposure, with the greater HP suppression during SPEAR task engagement for soldiers with high resilience. A larger sample would be required to test resilience as a mediator of psychological stress (e.g., casualty exposure) on autonomic flexibility and adaptability, but these data suggest that such a relationship is plausible. The study also suggests that simple interventions that could increase recovery of cardiac vagal tone after mentally taxing events, could also increase adaptability or improve recall of mission relevant information (i.e., the commander’s intent). The relationship between frequency of COs and autonomic reactivity to the posture challenge suggests that simple protocols could be designed to screen for cardiovascular signatures of psychological stress.

While it was hypothesized that the executive function tasks would represent a dimension of adaptability and therefore be significantly correlated with adaptability, performance on the executive function tasks was not predictive of performance on the SPEAR task. However, the challenges did lead to reduced vagal inhibitory impact on cardiac rate, and recovery of that inhibitory control following the executive function challenges (at FRB) was predictive of SPEAR performance. Recovery of vagal capacity was particularly predictive of performance on the SFA block, which proved significantly more difficult for soldier participants and which required non-kinetic, adaptive solutions. The strong correlation between the rate of RSA recovery and performance on the non-kinetic mission challenges of the SPEAR task suggests that inhibitory capacity is essential to the cognitive processes involved in adaptive problem solving on the modern battlefield. Convergent evidence observed in the EDA data at recovery (at FRB) indicated that declining sympathetic arousal was also predictive of performance on the SPEAR test task.

As expected, we did not find consistent or reliable evidence that HPA reactivity and regulation was associated with SPEAR task performance or executive function. The literature suggests that the HPA axis reacts to circumstances that are novel, when the demands of the situation are unfamiliar, and when individuals who find themselves in such circumstances do not have the capacity or previous experience to adapt to the situation. Given the prior military and leadership experience of this study’s participants, it is not surprising that we did not detect changes in cortisol reactivity and regulation in response to the SPEAR task. The “null” result here may be in fact an indirect indication of adaptability and resilience. Follow up studies that explore a more heterogenous sample and a broader range of challenges, both novel and familiar, will be required to establish this hypothesis.

We did expect that the change in sAA would be linked to engagement and active problem solving. We observed associations between sAA levels at specific time points (samples 4 and 5) during the SPEAR task that support this notion. Given these preliminary sAA-related findings, future examination of inter- and intraindividual differences in sAA in a larger sample and in the context of the SPEAR task could predict adaptability.

The combination of the RSA, EDA and sAA findings are supportive of the importance of the recovery response to adaptability. The vagal break appears to be the quickest means for the body to change arousal state to meet new and demanding situations. Rapid recovery of cardiac vagal tone shifts the autonomic systems back into normal balance, as at rest, so the participant is ready for the next problem set. SPEAR required cognitive shifting and agile removal of the vagal break and regaining vagal inhibition to support problem solving. Greater vagal capacity prior to the task appeared to maximize the engagement within and switching between the different demands of the SPEAR task.

Our initial notion of adaptability was constrained to concepts and responses associated with meeting the demands of a challenge. We now understand that the recovery from challenge is crucial to understanding adaptability and performance. The operational setting consists of multidimensional, complex, and often competing demands. The military leader is required to engage, distinguish relevant from irrelevant information, consider options, and refine or create new commands in a timely manner and often under conditions of high stress. Adaptive leadership is meeting such challenges time and again, and until the mission is accomplished. The flexible cardiac response identified in this study provides evidence of a functional and regulatory system response supportive of adaptive problem solving.

With the proper balance of arousal and recovery, individuals and units could better maintain high levels of battlefield effectiveness over extended periods of time. The SPEAR task provides a quantifiable basis on which to determine the optimal amount of rest and recovery needed by units and by individuals to facilitate sustainable adaptability. In addition, the metrics used in the present study could serve as the basis for evaluating supportive training strategies such as biofeedback that could target the autonomic balance directly.

Future work in this domain should include a test battery that taxes multiple processes of cognitive performance to include, for example, math computations, psychomotor vigilance, auditory discrimination, and working memory. Such a task would be expected to evoke a strong regulatory response in all subjects from which recovery would be highly predictive of adaptive problem solving. Beyond laboratory tasks that engage multiple and complex processes, we recommend the development of more authentic, mission relevant test tasks. Such an approach would bridge the current gap between experimentation and field-based training. To advance operational capability, research findings must be validated in large samples in order to address questions of scalability and determine methods to translate evidence-based findings to authentic, field-based training environments.

In order to better understand the generalizability of these finding to adaptability, we recommend extending the study of adaptability beyond the military. We recommend future efforts test a larger military sample representing soldiers across the rank and military occupation specialty (MOS) structure and a large civilian sample representative of the general population. Test tasks could include general cognitive workload, emotion regulation and physical stressors as well as occupational-relevant challenges in order to discern the different aspects of adaptability and confirm the specificity of the adaptability self-regulatory response.

The battlefield is not the only context where adaptive problem solving is critical. First responders, emergency medical teams, disaster relief personnel, fireman, law enforcement officers, and educators, for example, face job-related challenges replete with varying and uncertain performance demands and for which sustained adaptability is required for success. Natural disasters, terrorist attacks, and emergency situations produce the same physiological reactions, tax first responders and victims in the same manner, and require decision-making under stress similar to combat. These same metrics used for military adaptability apply to law enforcement and other stressful public service occupations as well.

These data highlight the importance of a certain response needed to meet the demands of the task and also the importance of recovery as critical to preparation for the next challenge. Military fitness and leader development programs can be developed to increase the recovery capacity of individuals regardless of the individual’s physical fitness capacity. Instead of a distinct physical training and task preparation focus, as is characteristic in today’s military, it is recommended to shift or augment the focus to psychophysiological training outcomes to achieve higher adaptability and consequently, resilience across the force. In this manner, soldiers would be both physiologically and psychologically equipped to meet the problem solving demands of the battlefield. Leader development programs should include activities that improve the psychophysiological capacity of junior leaders through problem solving and field-based training exercises that demand the pattern of arousal and recovery required for adaptive performance over time (i.e., sustainability). The military must connect the psychophysiological aspects of human performance with its training, education, and mission and battle preparations to optimize human performance in very ambiguous modern warfare that requires more thoughtfulness and less kinetic solutions. Understanding the psychophysiological correlates of battlefield stress could be leveraged to examine dose–response relationships and identify risk factors associated with combat-related disorders.

These indicators should also be incorporated into the military’s various selection and assessment programs designed to determine individuals most suited for high risk missions, high stress occupational specialties, and activities that require the most adaptability, such as aviation and special operations activities, and frontline leaders and commanders. This concept applies to any small unit that operates independently across a decentralized and non-contiguous battlefield. Training and advising foreign militaries in austere environments during conflict requires that junior leaders and small unit leaders have a high level of adaptability and resilience. The military would benefit from developing all of its junior leaders in this fashion, so decision-making is adaptable and decision-makers are adaptive and resilient.

In conclusion, these findings indicate flexible autonomic regulation supports recovery following challenge, which in turn supports problem solving or adaptability skills. The response/recovery parameters established in the present study could be applied to predict soldier problem solving as well as resilience to environmental stressors. Autonomic regulation can be enhanced through targeted cardiovascular training designed to increase cardiac vagal capacity. Both the executive function and SPEAR tasks engaged cardiovascular regulatory systems and thus provide a portal to investigate adaptive state regulation. Having identified the neural regulation and balance system associated with problem solving and adaptability, we can now apply these findings to performance enhancement. Cardiac regulation during the SPEAR task was related to psychological resilience, and the interaction between the components of adaptability, autonomic flexibility, and resilience suggests mental (and likely physical) health benefits would result from efforts to increase adaptability.

## Ethics Statement

This study was carried out in accordance with the recommendations of applicable Organizational, Local, State and Federal guidelines, as well as The Johns Hopkins Medicine Institutional Review Board (IRB) with written informed consent from all subjects. All subjects gave written informed consent in accordance with the Declaration of Helsinki. The protocol was approved by the Johns Hopkins Medicine IRB-X protocol #00068417 entitled “Soldier Performance and Effective, Authentic Response (SPEAR)” study. The Board determined that the device (BioPac System) was not significant risk (NSR) and meets the requirements for an abbreviated IDE. The IRB also determined that the project involved minimal risk to participants and that a Medical Monitor was not required under DoD Directive 3216.02. The IRB conducted scientific review per Organization Policy 111.5 and determined the research uses procedures consistent with sound research design and the research design is sound enough to yield the expected knowledge.

## Author Contributions

AH: PI, developed the study concept and protocol, implemented all aspects of the study, results preparation, dissemination, writing. GL: designed cardiovascular aspects of the protocol, ECG signal processing and analysis, results preparation, writing. MD: conducted ECG signal processing, analysis and results preparation. FW: supported SPEAR task development, data collection, signal processing, and results preparation. JG: supported SPEAR task design, data collection, lead the SPEAR task scoring team, interpretation, writing. CB: contributed to the salivary analytes analysis, results preparation, and interpretation. JK: contributed conceptual approach to the analysis and interpretation of HRV data, generated unique visualization of the RSA slope data. DG: contributed to the salivary analytes protocol design, analysis, interpretation, writing. WM: contributed to study implementation, data analysis and results preparation/interpretation, writing. All authors approved the manuscript and agreed to be accountable for all aspects of the work.

## Conflict of Interest Statement

All authors declare that the research was conducted in the absence of any commercial or financial relationships that could be construed as a potential conflict of interest. This was confirmed by AH, GL, MD, FW, JG, CB, JK, and WM. DG, however, provides the statement: “In the interest of full disclosure DAG is founder and chief scientific and strategy advisor at Salimetrics LLC and SalivaBio LLC. These relationships are managed by the policies of the committees on conflict of interest at Johns Hopkins University School of Medicine and the University of California at Irvine.”
